# *Piper retrofractum* Vahl. Extract, as a PPAR*δ* and AMPK Activator, Suppresses UVB-Induced Photoaging through Mitochondrial Biogenesis and MMPs Inhibition in Human Dermal Fibroblasts and Hairless Mice

**DOI:** 10.1155/2018/6172954

**Published:** 2018-02-12

**Authors:** Jungon Yun, Changhee Kim, Mi-Bo Kim, Jae-Kwan Hwang

**Affiliations:** Department of Biotechnology, College of Life Science and Biotechnology, Yonsei University, Seoul 03722, Republic of Korea

## Abstract

Photoaging occurs by UVB-irradiation and involves production of reactive oxygen species (ROS) and overexpression of matrix metalloproteinases (MMPs), leading to extracellular matrix damage.* Piper retrofractum* Vahl. is used as a traditional medicine for antiflatulence, expectorant, sedative, and anti-irritant; however, its antiphotoaging effect has not yet been studied. The current study investigated the antiphotoaging effect of standardized* Piper retrofractum* extract (PRE) on UVB-damaged human dermal fibroblasts and hairless mouse skin. PRE treatment activated the peroxisome proliferator-activated receptor delta (PPAR*δ*) and the adenosine monophosphate-activated protein kinase (AMPK), consequently upregulating mitochondrial synthesis and reducing ROS production. Additionally, PRE inhibited MMPs expression via suppressing mitogen-activated protein kinase (MAPK) and activator protein-1 (AP-1). PRE downregulated UVB-induced inflammatory reactions by inhibiting the nuclear factor-kappa B (NF-*κ*B) activity. PRE also enhanced transforming growth factor-beta (TGF-*β*) and the Smad signaling pathway, thereby promoting procollagen gene transcription. Furthermore, oral administration of PRE (300 mg/kg/day) similarly regulated the signaling pathways and increased antioxidant enzyme expression, thus attenuating physiological deformations, such as wrinkle formation and erythema response. Collectively, these results suggest that PRE acts as a potent antiphotoaging agent via PPAR*δ* and AMPK activation.

## 1. Introduction

Skin aging is divided into intrinsic aging and extrinsic aging. Intrinsic aging results from physiological changes that occur over time [1]. On the other hand, extrinsic aging is caused by external factors such as chemicals, stress, physical stimulation, or ultraviolet radiation [2]. As a major cause of photoaging, ultraviolet (UV) reaches to the upper-side of the dermis, inducing DNA damage, inflammation, and accumulation of reactive oxygen species (ROS) [3].

Mitochondria are organelles that regulate ATP biosynthesis and cell apoptosis [4]. Mitochondrial dysfunction is associated with various age-related degenerations [5]. In photoaging, UVB disrupts the mitochondrial electron transport system, resulting in mitochondrial dysfunction and apoptosis [6]. Maintenance of the number of mitochondria can delay the development of skin photoaging. Mitochondrial biogenesis, a process to form new mitochondria, is regulated by peroxisome proliferator-activated receptor gamma coactivator-1 alpha (PGC-1*α*), nuclear respiratory factor-1 (NRF-1), and mitochondrial transcription factor A (Tfam) [7]. Thus, increasing the number of mitochondria by upregulating these genes can be a way to prevent skin photoaging.

Peroxisome proliferator-activated receptor delta (PPAR*δ*), a member of the ligand-inducible PPAR nuclear receptor family, regulates target gene expression by forming a heterodimer with a retinoid X receptor and binding to a specific region in the promoter of target genes, called the peroxisome proliferator hormone response elements (PPRE) [8, 9]. PPAR*δ* participates in lipid oxidation, thermogenesis, energy uncoupling, and mitochondrial biogenesis [10, 11]. Adenosine monophosphate-activated protein kinase (AMPK) plays a central role in synthesizing mitochondria by activating PGC-1*α* [12]. Hence, both PPAR*δ* and AMPK can be targeted to maintain mitochondrial integrity in UVB-damaged skin.

Maintaining collagen and gelatin contents in the extracellular matrix (ECM) is important to prevent wrinkle development in skin that has been exposed to UVB. Matrix metalloproteinases (MMPs) are enzymes that degrade ECM components, such as collagen and gelatin. Secretion of MMPs is stimulated by UVB-irradiation induced activator protein-1 (AP-1) complex activation [13]. Another role of the AP-1 complex is to interrupt collagen synthesis by adversely affecting transforming growth factor-beta (TGF-*β*)/Smad signaling cascades [14]. Recently, PPAR*δ* activation was shown to upregulate collagen gene expression and suppress MMPs expression in UV-induced human dermal fibroblasts and keratinocyte [8, 9, 15]. Enhanced AMPK expression also suppresses MMP-1 expression in UVB-irradiated human dermal fibroblasts [16]. Thus, PPAR*δ* and AMPK attenuate wrinkle formation by the expression of regulating MMPs and collagen synthesis-related genes.

*Piper retrofractum *Vahl. (synonym* Piper officinarum *(Miq.) C DC) is mainly grown in tropical and subtropical regions [17]. The* P. retrofractum* fruit is used as a food spice and in traditional medicine as an antiflatulent, expectorant, antifungal, antitussive, sedative, and anti-irritant and for uterine contraction [18]. In a previous study,* P. retrofractum* extract (PRE) stimulated PPAR*δ* and AMPK activation in an obesity model [18]. Therefore, we hypothesized that PRE could confer an antiphotoaging effect via PPAR*δ* and AMPK activation. In this research, the antiphotoaging effect of PRE was evaluated in UVB-damaged human dermal fibroblasts and hairless mouse skin.

## 2. Materials and Methods

### 2.1. Preparation of Standardized PRE

The fruits of* P. retrofractum* were collected from Jakarta, Indonesia. A voucher specimen is deposited at the Department of Biotechnology, Yonsei University, Seoul, Korea. Dried fruit parts of* P. retrofractum* were ground and extracted with 99% ethanol in a shaker at 40°C for 4 h. The ethanol extract was filtered and the solvent was evaporated by a Laborota 4000 (Heidolph Instruments GmbH & Co. KG, Schwabach, Germany) with a yield of 6.8% (w/w). The standardized PRE included 2.8% (w/w) of dehydropipernonaline as a bioactive compound [18].

### 2.2. Reagents and Antibodies

For cell culture, Dulbecco's Modified Eagle's Medium (DMEM), fetal bovine serum (FBS), and antibiotic-antimycotic solution were purchased from Hyclone Laboratories Inc. (Logan, UT, USA), Gibco (Grand Island, NY, USA), and Wisent Inc. (Quebec, Canada), respectively. For Western blot, primary antibodies against PGC-1*α*, MMP-1, MMP-2, MMP-3, MMP-9, MMP-13, phospho(p)-extracellular signal-regulated kinase (ERK) ERK, p-c-Jun N-terminal kinase (JNK), JNK, p-p38, p38, p-c-Jun, c-Jun, c-Fos, nuclear factor-kappa B (NF-*κ*B), catalase, TGF-*β*, Smad2/3, and Smad7 were procured from Santa Cruz Biotechnology Inc. (Dallas, TX, USA). Primary antibodies against p-AMPK, AMPK, and *α*-tubulin were obtained from Cell Signaling Technology (Beverly, MA, USA). Secondary antibodies were purchased from Bethyl Laboratories (Montgomery, TX, USA).

### 2.3. Cell Culture

Monkey kidney (COS-7) and human dermal fibroblasts (HS68) were purchased from American Type Culture Collection (Manassas, VA, USA). Both cells were grown in DMEM with 10% FBS and 1% antibiotic-antimycotic solution in an atmosphere of 5% CO_2_ at 37°C.

### 2.4. Reporter Gene Luciferase Assay

COS-7 cells were transfected with pFA-PPAR*δ* and pFR-luciferase using Lipofector-EXT™ (AptaBio, Gyeonggi-do, Korea). After 4 h of transfection, the cells were stabilized with 10% FBS-DMEM for 24 h. Then, cells were treated with PRE and GW501516 (GW, Sigma-Aldrich, St. Louis, MO, USA) for an additional 24 h. The PPAR*δ* binding activity was assessed according to the luciferase assay system (Promega, Madison, WI, USA). Luciferase activity was determined using a MicroLumatPlus LB 96V Luminometer (Berthold Technologies GmbH & Co. KG, Bad Wildbad, Germany).

### 2.5. UVB-Irradiation

At 80% confluence, the medium was substituted with Dulbecco's Phosphate-Buffered Saline (DPBS) and HS68 cells were irradiated with UVB (15 mJ/cm^2^) using the UV crosslinker CL-1000M (UVP, Cambridge, UK). After washing with DPBS, the cells were treated with DMEM including PRE for an additional 24 h.

### 2.6. Mitochondrial Content Measurement

After UVB-irradiation and PRE treatment (0.5–5 *μ*g/mL), the HS68 cells were stained with 20 nM MitoTracker® Green FM (Invitrogen, Carlsbad, CA, USA) for 30 min. The mitochondrial levels were determined using a Gemini EM Microplate Reader (Molecular Devices, Sunnyvale, CA, USA) with excitation and emission wavelengths of 488 and 520 nm, respectively.

### 2.7. ROS Production Measurement

After UVB-irradiation and PRE treatment (0.5–5 *μ*g/mL), the HS68 cells were stained with 40 *μ*M 2′,7′-dichlorodihydrofluorescein diacetate (DCFH-DA; Sigma-Aldrich) for 30 min. ROS production was analyzed by Gemini EM Microplate Reader (Molecular Devices) with excitation and emission wavelengths of 488 nm and 520 nm, respectively.

### 2.8. Animal Experiments

Five-week-old female albino hairless mice (SKH-1) were purchased from Orient Bio Inc. (Gyeonggi-do, Korea). The experimental protocols were approved by the Institutional Animal Care and Use Committee of Yonsei Laboratory Animal Research Center (Permit number: 201509-471-03). Eighteen mice were randomly divided into three groups: (1) normal group, (2) UVB group, and (3) UVB + PRE group. For 8 weeks, mice of the UVB and the UVB + PRE groups were exposed to UVB every alternate day using the UV crosslinker CL-1000M (UVP). The UVB dose started at 75 mJ/cm^2^. Then, the dose was increased by 1 minimal erythema dose (MED) per week, until 3 MED, which was maintained until end of the experiments. Mice of the UVB + PRE group were orally given 300 mg/kg/day PRE and the remaining groups received saline. After 8 weeks, the mice were anesthetized using 2,2,2-tribromoethanol (Sigma-Aldrich) and sacrificed. For optical microscopy, the dorsal skin samples were fixed in 10% formalin and the residual dorsal skin was stored at −80°C.

### 2.9. Skin Surface Physiology for Wrinkle Measurement

The replica of mice dorsal skin was collected before sacrifice using replica full kit (Epigem, Seoul, Korea) and analyzed with Visioline VL 650 (CK Electronics GmbH, Cologne, Germany).

### 2.10. Histological Analysis

After sacrifice, the fixed skin samples were stained for collagen and skinfold thickness using Masson's trichrome (M&T) and hematoxylin and eosin (H&E), respectively. The stained skin samples were observed and photographed by an inverted microscope with twin CCD cameras (Eclipse TE2000-U, Nikon, Tokyo, Japan).

### 2.11. Hydroxyproline Assay

The amount of hydroxyproline in the dorsal skin was determined using the hydroxyproline assay kit (QuickZyme Biosciences, Leiden, Netherlands) according to the manufacturer's instructions.

### 2.12. Evaluation of Erythema Value

Before sacrifice, the erythema value was measured with a Mexameter® MX18 (CK Electronics GmbH).

### 2.13. Evaluation of Skinfold Thickness

The dorsal skin of mice was manually lifted by pinching between the neck and the base of tail. Skinfold thickness of the dorsal skin at the mid-back was measured with a caliper (Ozaki MFG Co., Ltd., Tokyo, Japan).

### 2.14. Western Blot Analysis

The proteins in HS68 cells and homogenized dorsal skin (approximately 1 × 1 cm^2^) were isolated using NP40 protein extraction solution (ELPIS-Biotech) containing 0.2% protease inhibitor cocktail (Sigma-Aldrich). The extracted proteins were equally quantified by Bradford assay (Bio-Rad, Hercules, CA, USA) and separated by sodium dodecyl sulfate-polyacrylamide gel electrophoresis (SDS-PAGE). The separated proteins were transferred onto a membrane (GE Healthcare, Piscataway, NJ, USA). After blocking the membrane with skim milk (5%) dissolved in Tris-buffered saline with Tween-20 (TBST), the membrane was incubated overnight at 4°C with primary antibodies. The membrane was washed with TBST, followed by incubation with secondary antibody for an additional 2 h. The membrane was visualized with ECL detection reagents (GE Healthcare, Piscataway, NJ, USA) using G:BOX Chemi XL (Syngene, Cambridge, UK).

### 2.15. Reverse Transcription-Polymerase Chain Reaction (RT-PCR) Analysis

Total mRNA was extracted from HS68 cells and homogenized skin, with Trizol reagent (Takara, Tokyo, Japan). The cDNA was synthesized with 2000 ng of extracted RNA and Reverse Transcription Premix (ELPIS-Biotech, Daejeon, Korea). Then the cDNA was amplified with each primer and HiPi Premix (ELPIS-Biotech). The primer sequences were as follows: human NRF-1 (forward, 5′-AGTTCAAAAG ATGAAGGACA-3′; reverse, 5′-GTTTGCCTGCTGTGATGTGG-3′), human Tfam (forward, 5′-AGCTCAGAACCCAGATGCAA-3′; reverse, 5′-TTCAGCTTTTCCTGCGGTGA-3′), human ERR*α* (forward, 5′-ATGGTGTGGCATCCTGTGAG-3′; reverse, 5′-ATTCACTGGGG CTGCTGTC-3′), human COL1A1 (forward, 5′-CACGACAAAGCAGAAACATC-3′; reverse, 5′-ACACATCAAGACAAGAACGAG-3′), human COL3A1 (forward, 5′-TGGTGCCCCTGG TCCTTGCT-3′; reverse, 5′-TACGGGGCAAAACCGCCAG C-3′), human COL4A1 (forward, 5′-TCCTGGCCTCCAGGGAATTA′-3′; reverse, 5′-ATCAACAGATGGGGTGCCTG-3′), human COL7A1 (forward, 5′-CTGGGAGAGAAGGTCGTGATGG-3′; reverse, 5′-TCCACAT TCGGCACACTTCC-3′), human interleukin- (IL-) 1*β* (forward, 5′-AGCCATGGCAGAAGTACCTG-3′; reverse, 5′-TCCATGGCCCACAACAACTGA-3′), human IL-6 (forward, 5′-ATGAGGAGAC TTGCCTGGTG-3′; reverse, 5′-ACAACAATCTGAGGTGCCCA-3′), human IL-8 (forward, 5′-CCAGGAAGAAACCACCGGAA-3′; reverse, 5′-CCTCTGCACCCAGTTTTCCT-3′), human GAPDH (forward, 5′-CTCCTGTTCGACAGTCAGCC-3′; reverse, 5′-TCGCCCCACT TGATTTTGGA-3′), mouse COL1A1 (forward, 5′-GTCCCCAATGGTGAGACGTG-3′; reverse, 5′-GCACGGAAACTCCAGCTGAT-3′), mouse COL3A1 (forward, 5′-AGCGGCTG AGTTTTATGACG-3′; reverse, 5′- AGCACAGGAGCAGGTGTAGA-3′), mouse COL4A1 (forward, 5′-GCCAAAGCCAAACCCATTCC-3′; reverse, 5′-TGGTACGTGTGGTAACTTC TC-3′), mouse COL7A1 (forward, 5′-AAGCCGAGATTAAGGGCTGG-3′; reverse, 5′-CACC AAATGGAGCACAGCAG-3′), mouse *β*-actin (forward, 5′-GCTCCGGCATGTGCAA-3′; reverse, 5′-AGGATCTTCATGAGGTAGT-3′). The entire process was conducted on a Gene Amp PCR System 2700 (Applied Biosystems, Foster City, CA, USA). The amplified cDNA was separated by electrophoresis and verified by G:BOX Chemi XL (Syngene).

### 2.16. Statistical Analysis

Data are presented as the mean ± standard deviation (SD). Statistical analyses were performed with SPSS 23.0 (SPSS Inc., Chicago, IL, USA) using one-way analysis of variance (ANOVA) followed by Scheffe's test. ^*∗*^*p* < 0.05, ^*∗∗*^*p* < 0.01, and ^##^*p* < 0.01 were considered statistically significant.

## 3. Results

### 3.1. The Effects of PRE on PPAR*δ* Binding Activity, Mitochondrial Biogenesis, and ROS Accumulation* In Vitro*

**Figure 1:**
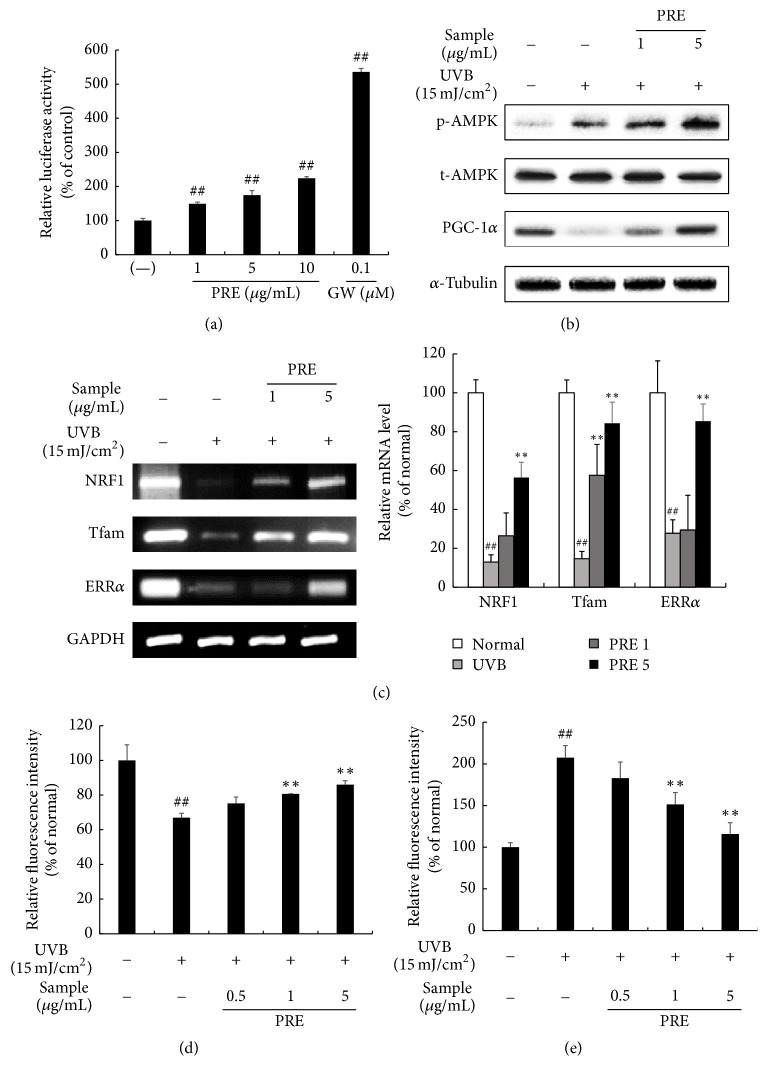
The effects of PRE on the PPAR*δ* activity, mitochondrial biogenesis, and ROS accumulation* in vitro*. (a) The PPAR*δ* agonistic activity of PRE was measured by the PPAR*δ*-luciferase transactivation assay. GW501516 was used as a positive control. (b) The expression of AMPK and PGC-1*α* was evaluated by Western blot. (c) The expression of mitochondrial biogenesis-related genes was evaluated by RT-PCR. Equal protein and mRNA loading were verified by *α*-tubulin and GAPDH, respectively. (d) The mitochondrial content was analyzed by MitoTracker. (e) The ROS content was measured by DCFH-DA. Data are represented as the mean ± SD from triplicate independent experiments. ^##^*p* < 0.01 compared to normal; ^*∗∗*^*p* < 0.01 compared to UVB.

To investigate whether PRE increased PPAR*δ* binding activity, we performed reporter gene luciferase assay using GW, a well-known PPAR*δ* agonist [8] as a positive control. PRE treatment activated PPAR*δ* binding activity in a dose-dependent manner (Figure 1(a)). We verified AMPK activation by treating UVB-damaged HS68 cells with PRE. UVB-irradiation slightly activated p-AMPK; however, PRE dose-dependently activated p-AMPK (Figure 1(b)). In order to confirm the mitochondrial biogenesis by PRE, we evaluated the mitochondrial biogenesis signaling pathway, PGC-1*α*, Tfam, NRF1, and ERR*α*. This signaling pathway was suppressed by UVB-irradiation, but PRE restored these markers (Figures 1(b) and 1(c)). The mitochondrial content was decreased to 67% by UVB-irradiation; however, PRE significantly restored the mitochondrial content (Figure 1(d)). ROS accumulation in the fibroblasts was enhanced to 207% by UVB-irradiation but attenuated by PRE treatment (Figure 1(e)).

### 3.2. The Effects of PRE on MMPs and Inflammatory Cytokines Expression* In Vitro*

**Figure 2:**
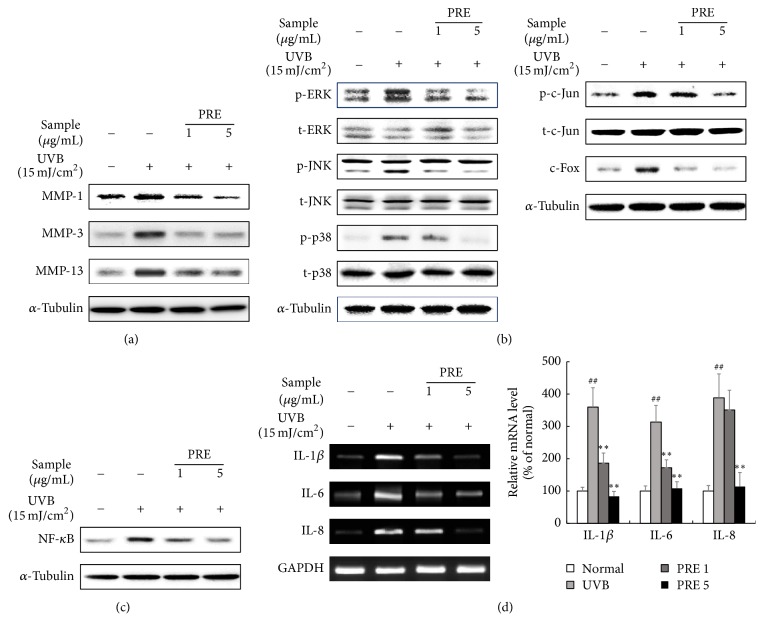
The effects of PRE on MMPs expression and its mechanisms* in vitro*. (a) The MMPs protein expression was evaluated by Western blot. (b) The expression of MAPKs and AP-1 was investigated by Western blot. (c) The expression of NF-*κ*B was assessed by Western blot. (d) The expression of inflammatory cytokines was determined by RT-PCR. Equal protein and mRNA loading were identified by *α*-tubulin and GAPDH, respectively. Data are represented as the mean ± SD from triplicate independent experiments. ^##^*p* < 0.01 compared to normal; ^*∗∗*^*p* < 0.01 compared to UVB.

In human dermal fibroblasts, the protein expression levels of MMP-1, MMP-3, and MMP-13 were stimulated by UVB-irradiation, whereas PRE ameliorated the MMPs expression (Figure 2(a)). UVB-induced phosphorylation of mitogen-activated protein kinases including ERK, JNK, and p38 was decreased in the PRE treated human dermal fibroblasts (Figure 2(b)). UVB-irradiation enhanced the expression levels of AP-1 complex, p-c-Jun, and c-Fos. PRE treatment suppressed the expression of AP-1 complex in a dose-dependent manner (Figure 2(b)). Furthermore, UVB-irradiation also induced the expression of inflammatory factors, such as NF-*κ*B, IL-1*β*, IL-6, and IL-8, whereas PRE treatment inhibited their expression levels (Figures 2(c) and 2(d)). These results indicate that PRE inhibits the expression of MMPs by suppressing the MAPKs/AP-1 pathways and protects the skin from UVB-induced inflammation by attenuating NF-*κ*B expression.

### 3.3. The Effect of PRE on Collagen Synthesis* In Vitro*

**Figure 3:**
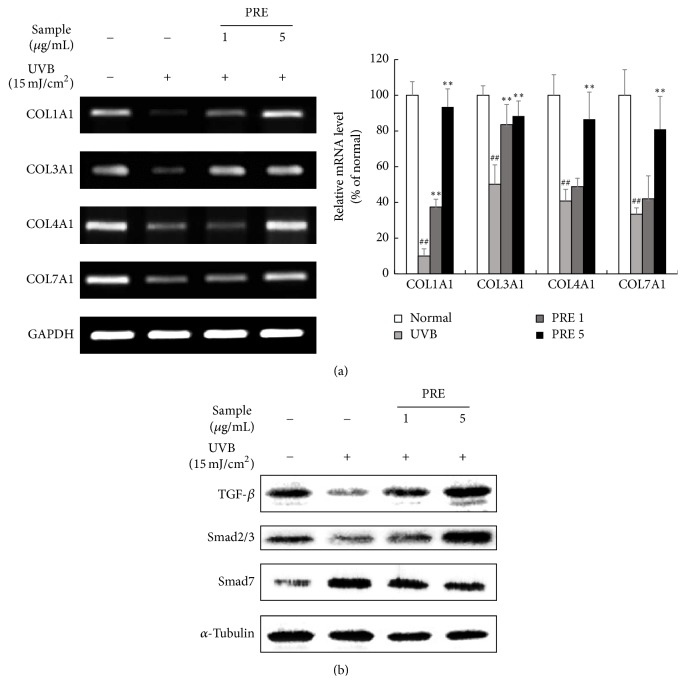
The effects of PRE on collagen gene transcription and its mechanism* in vitro*. (a) The expression of collagen genes was investigated by RT-PCR. (b) The expression of TGF-*β*/Smad signaling was measured by Western blot. Equal mRNA and protein loading were identified by GAPDH and *α*-tubulin, respectively. Data are represented as the mean ± SD from triplicate independent experiments. ^##^*p* < 0.01 compared to normal; ^*∗∗*^*p* < 0.01 compared to UVB.

The mRNA expression levels of COL1A1, COL3A1, COL4A1, and COL7A1 were reduced by UVB-irradiation, but PRE treatment markedly increased these genes expression in human dermal fibroblasts (Figure 3(a)). TGF-*β* and Smad2/3 stimulate the collagen synthesis, but Smad7 acts as a TGF-*β* antagonist [19]. The protein expression of TGF-*β* and Smad2/3 was inhibited in response to UVB-irradiation, while that of Smad7 was upregulated. After PRE treatment, TGF-*β* and Smad2/3 were overexpressed and Smad7 was suppressed (Figure 3(b)).

### 3.4. The Effect of PRE on UVB-Irradiated Skin Physiological Changes* In Vivo*

**Figure 4:**
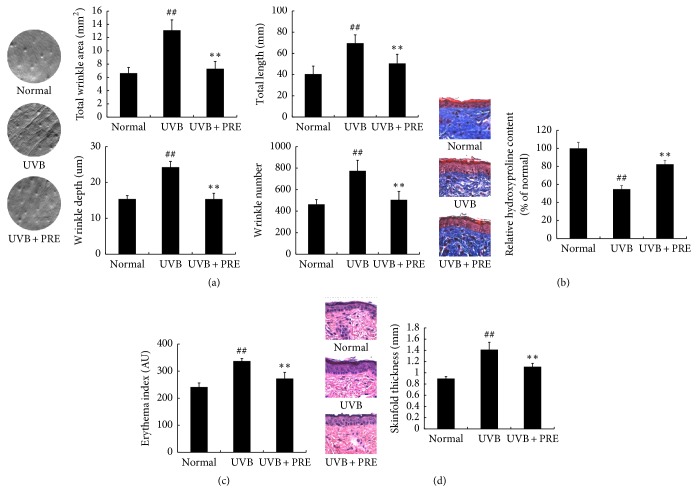
The effect of PRE on skin physiology. (a) The replica of the mice dorsal skin was analyzed. (b) Collagen fiber in the skin was observed by staining with Masson's trichrome stain. Hydroxyproline content in skin sample was estimated by a hydroxyproline kit. (c) Skin erythema value was investigated by Mexameter. (d) Skinfold thickness in the skin was observed by staining with hematoxylin and eosin. Skinfold thickness was measured with a caliper. Data are expressed as the mean ± SD of six mice per group. ^##^*p* < 0.01 compared to normal; ^*∗∗*^*p* < 0.01 compared to UVB.

The wrinkles replica was quantitatively analyzed based on its number, depth, length, and total area. Wrinkle development in the UVB-damaged skin of mice decreased in the UVB + PRE group compared to that in the UVB group (Figure 4(a)). In order to identify the changes in collagen content caused by UVB-irradiation, the stained skin sections and hydroxyproline contents were assessed. Collagen and hydroxyproline contents were diminished in the UVB-damaged skin of hairless mice, whereas the UVB + PRE group showed increased collagen and hydroxyproline contents (Figure 4(b)). As one of the inflammatory responses, erythema and skinfold thickness were suppressed by 19% and 20%, respectively, in the UVB + PRE group compared to that in the UVB group (Figures 4(c) and 4(d)). These results indicate that PRE attenuates wrinkle formation and erythema in photoaging animal model.

### 3.5. The Effects of PRE on Mitochondrial Biogenesis and Antioxidant Enzyme, MMPs, and NF-*κ*B Expression* In Vivo*

**Figure 5:**
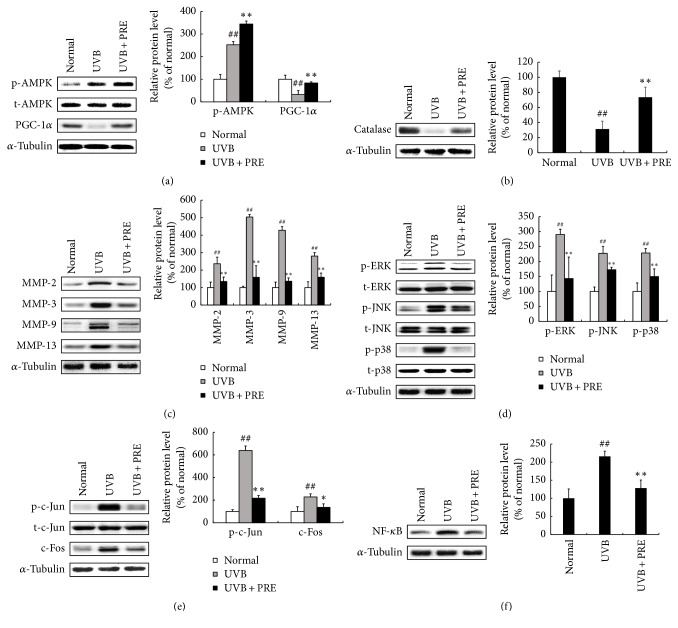
The effects of PRE on mitochondrial biogenesis, MMPs expression, an antioxidant enzyme, and NF-*κ*B* in vivo*. (a) The expression of AMPK and PGC-1*α* was investigated by Western blot. (b) The expression of catalase was assessed by Western blot. (c) The expression of MMPs was measured by Western blot. (d) The expression of MAPKs was investigated by Western blot. (e) The AP-1 complex components were determined by Western blot. (f) The expression of NF-*κ*B was evaluated by Western blot. Equal protein loading was identified by *α*-tubulin. Data are expressed as the mean ± SD of six mice per group. ^##^*p* < 0.01 compared to normal; ^*∗*^*p* < 0.05 and ^*∗∗*^*p* < 0.01 compared to UVB.

In the animal model, UVB-irradiation increased p-AMPK but decreased PGC-1*α*. Treatment of PRE significantly upregulated p-AMPK and PGC-1*α* compared to the UVB group (Figure 5(a)). The expression of catalase, an antioxidant enzyme, was reduced by 32%, but PRE treatment enhanced its expression by 74% (Figure 5(b)). UVB-irradiation increased the protein expression levels of MMP-2, MMP-3, MMP-9, and MMP-13 by 237, 504, 428, and 280%, respectively, whereas PRE treatment reduced the MMPs expression compared to that in the UVB group (Figure 5(c)). The MAPK components, ERK, JNK, and p38, were phosphorylated by about 249%, yet the group that received PRE showed an average suppression of 36% compared to the UVB group (Figure 5(d)). The p-c-Jun and c-Fos expression levels were reduced by 66 and 41%, respectively, in the UVB + PRE group compared to those in the UVB group (Figure 5(e)). NF-*κ*B expression was induced by 216% in the UVB group; however, PRE supplementation suppressed NF-*κ*B expression by 41% compared to the UVB group (Figure 5(f)).

### 3.6. The Effect of PRE on Collagen Synthesis* In Vivo*

**Figure 6:**
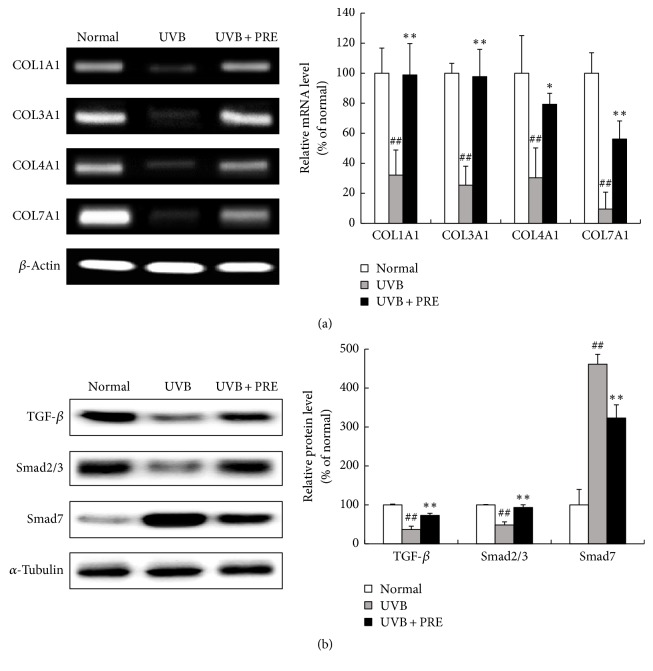
The effects of PRE on collagen gene expression and its mechanisms* in vivo*. (a) The expression of collagen genes was determined by RT-PCR. (b) The expression of TGF-*β*/Smad was evaluated by Western blot. Equal mRNA and protein loading were identified by *β*-actin and *α*-tubulin, respectively. Data are expressed as the mean ± SD of six mice per group. ^##^*p* < 0.01 compared to normal; ^*∗*^*p* < 0.05 and ^*∗∗*^*p* < 0.01 compared to UVB.

The mRNA expression levels of COL1A1, COL3A1, COL4A1, and COL7A1 in the UVB group were downregulated by 32, 25, 30, and 9.5%, respectively. The PRE administrated group induced the mRNA levels of the collagen genes at 208%, 284%, 161%, and 439%, respectively, compared to those in the UVB group (Figure 6(a)). In the UVB + PRE group, the collagen synthesis-related genes (TGF-*β* and Smad2/3) were upregulated, whereas Smad7 expression was suppressed by 30% compared to that in the UVB group (Figure 6(b)).

## 4. Discussion

PPAR*δ* and AMPK activation participate in the mitochondrial biogenesis in several organs [20, 21]. Cotreatment with GW1516, a PPAR*δ* agonist, and 5-aminoimidazole-4-carboxamide ribonucleotide (AICAR), an AMPK agonist, has a synergistic effect on endurance related gene transcription in the muscle [22], which could be closely involved in mitochondrial function. The PRE fraction activates PPAR*δ* and AMPK in high-fat diet-induced obese mice [18]. Consistently, PRE also activated PPAR*δ* and AMPK in the UVB-damaged human dermal fibroblasts and skin of hairless mice, thereby promoting mitochondrial biogenesis (Figures 1(a)–1(d)). However, this study showed that UVB itself alleviated p-AMPK expression but did not restore mitochondrial biogenesis (Figures 1(b)–1(d)). This may be a result of destructive activity of dysfunctional mitochondria caused by UVB-induced AMPK activation [23]. On a molecular level, p-AMPK increases unc-51 like autophagy activating kinase 1 (ULK1) translocation in dysfunctional mitochondria, leading to the destruction of mitochondria [24]. UVB-induced AMPK activation could be responsible for the disruption of mitochondria, but it is not sufficient for mitochondrial biogenesis. In our study, PRE increased the mitochondrial content in UVB-damaged dermal fibroblasts (Figure 1(d)). A possible explanation for this result is that PRE not only increased more AMPK activation than UVB, but also enhanced PPAR*δ* activity (Figures 1(a)–1(c)).

In UVB-damaged skin, oxidative stress occurs due to a decrease in the level of antioxidant enzymes, as well as disruption of the mitochondrial membrane [25]. Natural compounds and plant extracts, such as ellagic acid,* Agastache rugosa* extract, and* Foeniculum vulgare* extract, exhibited antiphotoaging effects by enhancing antioxidant enzymes [26–28]. Previously, the antioxidants effect of PRE was reported based on the 2,2-diphenyl-1-picrylhydrazyl (DPPH) radical scavenging assay and Folin-Ciocalteu method [29–31]. In the current study, PRE scavenged ROS in fibroblasts and upregulated the catalase expression level in hairless mice (Figures 1(e) and 5(b)). Thus, PRE inhibited the development of photoaging by increasing the expression of antioxidant enzymes and reducing oxidative stress.

UVB-irradiation-induced phosphorylation of the MAPKs triggers the activation of AP-1 components, consequently leading to expression of MMPs [3]. PRE, as a PPAR*δ* activator, inhibited the MMPs expression by downregulating MAPKs and AP-1 complex (Figure 2(b)). However, PPAR*δ* agonist is known to inhibit the AP-1 complex by selective p-JNK suppression [15]. This leads us to consider that AMPK is involved in regulating the signaling of MAPKs. AMPK agonist inhibits p-ERK expression in phenylephrine-induced p-ERK signaling [32] and p-p38 expression in palmitate-induced oxidative stress [33]. In addition, AMPK agonist participates in MMP-1 suppression in the UV-damaged human dermal fibroblasts [16], which could be related to MAPKs signaling. Taken together, PRE suppressed not only p-JNK, by PPAR*δ* activation, but also p-ERK and p-p38, through AMPK activation. MAPKs activation also interrupts TGF-*β*/Smad signaling cascades [14]. Moreover, PPAR*δ* agonist induces collagen synthesis via TGF-*β* activation in human dermal fibroblasts and aged rat skin [9, 34]. PRE, as a PPAR*δ* and AMPK activator, increased collagen synthesis by enhancing the TGF-*β*/Smad2/3 signaling pathway (Figures 3(b) and 6(b)) and suppressing MAPKs expression (Figures 2(b) and 5(d)). The combinatorial effects of increased collagen synthesis and inhibition of MMPs, by suppressing MAPKs, were major mechanisms in preventing histological deformations, such as wrinkle formation and alterations of the ECM structure in hairless mice (Figures 4(a) and 4(b)).

UVB-induced inflammatory responses are responsible for various physiological deformations, such as MMPs accumulation, irritation, and erythema [35]. There is a controversial relation between PPAR*δ* and inflammation. Some studies revealed that PPAR*δ* activators cause inflammation in the UVB-damaged skin through TGF-*β*1 mediated responses [36] and psoriasis in the mouse skin [37]. In contrast, another study showed that PPAR*δ* activator reduces inflammatory responses by suppressing NF-*κ*B [38]. In the present study, PRE, as a PPAR*δ* activator, downregulated NF-*κ*B expression, as well as the expression of the inflammatory cytokines, IL-1*β*, IL-6, and IL-8, in human dermal fibroblasts and hairless mouse skin (Figures 2(c), 2(d), and 5(f)). The erythema response was also relieved in PRE administrated mice (Figure 4(c)). PRE acted as a PPAR*δ* agonist and its inhibitory effect on NF-*κ*B activity was a major reason for attenuating inflammatory responses, which is in accordance with the finding to a previous study [38].

## 5. Conclusions

In the present study, PRE showed antiphotoaging activities in UVB-damaged human dermal fibroblasts and skin of hairless mice via PPAR*δ* and AMPK agonist activities. As a PPAR*δ* and AMPK activator, PRE induced mitochondrial biogenesis that was suppressed by UVB-irradiation. PRE also induced antioxidant enzyme expression. These effects by PRE treatment significantly attenuated ROS production. Additionally, PRE suppressed MMPs expression through PPAR*δ*/AMPK activation and MAPKs/AP-1 inhibition, while enhancing collagen genes expression by upregulating TGF-*β* and Smad signaling mediated by PPAR*δ* activation. PRE alleviated inflammatory responses by suppressing NF-*κ*B activity. PRE prevented histological deformations, like wrinkle formation, ECM destruction, and erythema mediated by UVB-irradiation in the skin of hairless mice. Taken together, these results suggested the potent antiphotoaging effects of PRE as a PPAR*δ* and AMPK activator, implying its potential use as a food ingredient for functional foods or nutraceuticals.
